# High rates of undiagnosed and uncontrolled hypertension upon a screening campaign in rural Rwanda: a cross-sectional study

**DOI:** 10.1186/s12872-022-02606-9

**Published:** 2022-04-26

**Authors:** Evariste Ntaganda, Regine Mugeni, Emmanuel Harerimana, Gedeon Ngoga, Symaque Dusabeyezu, Francois Uwinkindi, Jean N. Utumatwishima, Eugene Mutimura, Victor G. Davila-Roman, Kenneth Schechtman, Aurore Nishimwe, Laurence Twizeyimana, Angela L. Brown, W. Todd Cade, Marcus Bushaku, Lisa de Las Fuentes, Dominic Reeds, Marc Twagirumukiza

**Affiliations:** 1 Rwanda Biomedical Center (RBC), Rwanda Ministry of Health, Kigali, Rwanda; 2 Rwamagana Provincial Hospital, Rwamagana, Eastern Province Rwanda; 3 Partners in Health (PIH)/Inshuti Mu Buzima, Rwinkwavu, Rwanda; 4 National Council for Science and Technology (NCST), Kigali, Rwanda; 5 Cardiovascular Division, Department of Medicine, Washington University in St. Louis, St. Louis, MO USA; 6 Division of Biostatistics, Washington University in St. Louis, St. Louis, MO USA; 7 Regional Alliance for Sustainable Development (RASD Rwanda), Kigali, Rwanda; 8 Duke University School of Medicine, Durham, NC 27710 USA; 9 School of Medicine and Pharmacy, College of Medicine and Health Sciences, University of Rwanda, Kigali, Rwanda; 10 Faculty of Medicine and Health Sciences, Ghent University, Ghent, Belgium

**Keywords:** High blood pressure, Hypertension, Screening, Rwanda

## Abstract

**Background:**

Hypertension remains the major risk factor for cardiovascular diseases (CVDs) worldwide with a prevalence and mortality in low- and middle-income countries (LMICs) among the highest. The early detection of hypertension risk factors is a crucial pillar for CVD prevention.

**Design and method:**

This cross-sectional study included 4284 subjects, mean age 46 ± 16SD, 56.4% females and mean BMI 26.6 ± 3.7 SD. Data were collected through a screening campaign in rural area of Kirehe District, Eastern of Rwanda, with the objective to characterize and examine the prevalence of elevated blood pressure (BP) and other CVD risk factors. An adapted tool from the World Health Organization STEPwise Approach was used for data collection. Elevated BP was defined as ≥ 140/90 mm/Hg and elevated blood glucose as blood glucose ≥ 100 mg/dL after a 6-h fast.

**Results:**

Of the sampled population, 21.2% (n = 910) had an elevated BP at screening; BP was elevated among individuals not previously known to have HTN in 18.7% (n = 752). Among individuals with a prior diagnosis of HTN, 62.2% (n = 158 of 254) BP was uncontrolled. Age, weight, smoking, alcohol history and waist circumference were associated with BP in both univariate analyses and multivariate analysis.

**Conclusion:**

High rates of elevated BP identified through a health screening campaign in this Rwandan district were surprising given the rural characteristics of the district and relatively low population age. These data highlight the need to implement an adequate strategy for the prevention, diagnosis, and control of HTN that includes rural areas of Rwanda as part of a multicomponent strategy for CVD prevention.

**Supplementary Information:**

The online version contains supplementary material available at 10.1186/s12872-022-02606-9.

## Background

Cardiovascular diseases (CVDs) are the leading cause of morbidity and mortality worldwide [1, 2] and approximately 80% of all cardiovascular deaths occur in low- and middle-income countries [1, 3]. By 2030, cardiovascular deaths are projected to increase to 23 million globally [4] and double in Sub-Saharan Africa (SSA) from 1 million deaths in 2013 [5].

Hypertension (HTN) is the most prevalent risk factor for CVD mortality worldwide [3]. Although hypertension is a well-document public health threat in developing countries, population-based data on HTN prevalence is scarce from SSA. Based on one report, the estimated prevalence of HTN in SSA is 16.2%, ranging from 10.6% in Ethiopia to 29.6% in Ghana. In SSA, the prevalence of HTN is also higher in urban (20.7%) compared with rural (13.7%) areas [6], a difference generally attributed to lifestyle changes commonly associated increasing urbanization (physical inactivity, obesity, smoking, and alcohol consumption) [7]. Similar to other LMICs, Rwanda is experiencing epidemiological transition in urban areas with changes in lifestyle behaviors which portends increased healthcare burden, morbidity, and mortality from CVDs.

Early detection and management remain a key cost-effective strategy for the prevention of chronic CVDs [6, 8, 9]. However, there is a lack of data on the prevalence of CVD risk factors among adults living in remote rural areas which are less geographically accessible. This fundamental data gap hampers efforts to characterize the CVD risk profile in mainly rural regions, further impeding efforts to establish appropriate preventive measures targeting these underserved populations [1, 10]. To bridge this gap, this study aimed to measure the rates of and risk factors for HTN in people living in a rural area of Rwanda through a screening campaign the Kirehe District, an eastern province of Rwanda.

## Methods

### Study design and procedures

This was a cross-sectional study that included adult (≥ 18 years) Rwandan people who participated in campaign on voluntary basis for five days from 23rd to 29th September, 2018. The study design followed the other reported screening studies design [11]. In addition, it has integrated elements from Health Beliefs Model (HBM) [12] to explore the rates and predictors of HTN in the community. The screening was conducted by trained volunteers (medical students and nurses) from health centers (HCs) in the Kirehe district hospital catchment area. The core role of the Kirehe district leadership was to arrange with local authorities to sensitize and invite people for screening by using local and public communications and radio publicity spots. The screening site was designed by HCs leadership and staff of Rwanda Biomedical Centre (RBC), a government institution under the Ministry of Health responsible for implementing health polices and health services in the community.

### Study setting

The study was conducted in 8 sectors in the Kirehe District that were systematically selected. The Kirehe District, one of the rural area districts situated in Eastern province of Rwanda bordering Tanzania, covers a total area of 1118.5 Km^2^ with a population of 340,983 (52% female) according to National Institute of Statistics of Rwanda (NISR). Ninety percent (90%) of the economic activity in the district depends on agriculture and livestock [13].

### Data collection

Data collection was done after verbal consent of participants by using an adapted World Health Organization STEPwise Approach to NCD Risk Factor Surveillance tool [14]. Data collected included demographics, social history of alcohol intake and smoking, prior history of hypertension, and time since last meal. Measurements included anthropometrics, blood pressure and blood glucose. After a resting in a seated position for 5 min, blood pressure (BP) was taken three times separated by 1 min following a standardized protocol with a calibrated and automated BP machine (Omron M2, Kyoto, Japan). The mean of the 2nd and 3rd readings was recorded. Waist circumference (WC) was measured in triplicate at the level of the midpoint between the lower margin of the last palpable rib and the top of the iliac crest after expiration using a 203 cm Seca^®^ measuring tape (all Seca GmbH & Co. KG., Hamburg, Germany); the mean of the three WC measures was used for analysis [15]. Weight was measured using a digital Seca^®^ 813 scale and height using a Seca^®^ 213 stadiometer. As the BP measures were taken casually and do not necessarily reflect a well-established hypertension diagnosis [16], we used the term of “Elevated BP” to reflect a systolic BP greater than or equal to 140 mmHg and/or a diastolic BP greater than or equal to 90 mmHg [17, 18]. This definition of elevated BP has nothing do with the definition of hypertension staging, used in some guidelines [16]. Data on the use of anti-hypertensive medications was not collected; a separate question surveyed whether the subject had ever been diagnosed with HTN.

A screening blood glucose (BG) was measured using a portable-battery driven Accu-Check^®^ Aviva (Roche Diagnostics GmbH, Mannheim, Germany); the number of fasting hours as recorded and categorized form 0–6 h and 6 h and above (6 h corresponding to the night period from midnight to 6.00 a.m. where no food intake is usually expected). This was decided after the pilot where we found most of participant not remembering the exact time of their last food intake (not only food or meal but also including all kinds of mouth intake, a common situation in rural families).

As the BG measures were taken casually and do not necessarily reflect a well-established diabetes diagnosis [19], we used the term “elevated blood glucose” to reflect glycaemia greater than or equal to 100 mg/dL [19–21]. Participants who had elevated BP or self-reported use of HTN medications or had elevated BG were referred to the nearest health center for regular follow-up. Those individuals with normal BP were counselled on NCD risk reduction for primary prevention. The blood glucose and blood pressure results were communicated to participants immediately. The study was conducted in accordance with the principles of the Declaration of Helsinki and local Rwandan regulations.

### Statistical methods and data analysis

Data was captured by a study team person using spreadsheet software program (Microsoft Excel, Microsoft Corporation, 2018), then de-identified and transferred to STATA 15.5 Version (Stata Corporation, College Station, TX) for analysis. Data are presented as mean ± standard deviation (SD) or percentage (number). Comparisons were performed by unpaired t-tests, one-way ANOVA and χ^2^-test as appropriate. Results with *p* values < 0.05 were considered statistically significant. Univariate and multivariate regression analyses were used to assess relationships between different risk factors and the presence of elevated BP in the total population and in subgroups defined by the absence or presence of a prior diagnosis of HTN.

We inspect the correlations between the predictors, using a correlation map to assess the collinearity, and highly correlated variables were entered one by one to test the best model. All variables in bivariate analyses with *p* value < 0.05 were considered for inclusion in multivariate regression model.

## Results

### Participant characteristics by blood pressure category

A total of 4284 participants in Kirehe district completed a 5-day screening program as part of routine monthly physical activity national program. The participants were largely middle-aged (mean age: 46 ± 16 years, range: 18–98 years), female (56, 4%), mildly overweight, and the majority had a history of alcohol intake (Table 1). Females were younger than males (mean age: 45 ± 16 vs. 47 ± 16 years; *p* < 0.001). More than one-third of participants (34%) reported history of smoking; 14.8% (n = 636) were current smokers and 19.2% (n = 821) were former smokers.

**Table 1:** Participants’ characteristics and elevated blood pressure (BP) outcome

Participant characteristics	Total screened population N = 4284	Comparisons by elevated blood pressure^a^						
		Normal BP n = 3374	All elevated BP n = 910 (21.2%)		Elevated BP in known hypertensive subjects n = 158 (62.2%) of 254		Elevated BP in non-known hypertensive subjects n = 752 (18.7%) of 4030	
			Mean ± SD or % (n)	*p* value^b^	Mean ± SD or % (n)	*p *value^b^	Mean ± SD or % (n)	*p *value^b^
Female (%) (n)	56.4 (2417)	57.0 (1922)	54.4 (495)	0.165	54.4 (86)	0.530	54.4 (409)	0.197
Age (years)	46 ± 16	44 ± 15	55 ± 15	< 0.001	56 ± 14	< 0.001	55 ± 15	< 0.001
Height (cm)	166 ± 4	166 ± 4	166 ± 4	0.486	166 ± 4	0.193	166 ± 4	0.180
Weight (kg)	73.6 ± 9.3	72.8 ± 8.2	76.7 ± 12	< 0.001	76.2 ± 12.1	< 0.001	76.7 ± 12	< 0.001
BMI (kg/m^2^)	26.6 ± 3.7	26.4 ± 3.3	27.7 ± 4.6	< 0.001	27.8 ± 4.7	< 0.001	27.7 ± 4.7	< 0.001
Waist circumference (cm)	74.4 ± 16.6	72.7 ± 15.6	80.4 ± 18.6	< 0.001	80.0 ± 18.2	< 0.001	80.5 ± 18.6	< 0.001
Smoking history (%) (n)	34.0 (1457)	26.6 (896)	61.6 (561)	< 0.001	61.4 (97)	< 0.001	61.7 (464)	< 0.001
Alcohol history (%) (n)	63.8 (2732)	58.1 (1959)	84.9 (773)	< 0.001	82.9 (131)	< 0.001	85.4 (642)	< 0.001
Systolic BP (mmHg)	127 ± 18	119 ± 12	153 ± 14	< 0.001	153 ± 16	< 0.001	153 ± 13	< 0.001
Diastolic BP (mmHg)	76 ± 11	72 ± 8	88 ± 9	< 0.001	89 ± 9	< 0.001	88 ± 9	< 0.001
Blood glucose (mg/dL)	101 ± 22	100 ± 21	105 ± 25	< 0.001	103 ± 30	0.051	105 ± 24	< 0.001
Elevated blood glucose (%) (n)^c^	18.2 (779)	16.5 (558)	24.3 (221)	< 0.001	7.6 (12)	0.003	27.8 (208)	< 0.001

Table combine both descriptive data on the total screened population (first column) and univariate participants characteristics comparison between each time the normal BP group versus elevated BP, versus poorly controlled BP and versus new discovered cases BP groups

^a^Elevated blood pressure was defined as a systolic blood pressure (SBP) ≥ 140 mmHg and/or diastolic blood pressure (DBP) ≥ 90 mmHg

^b^Comparison by unpaired t-test or χ^2^, Normal BP as comparison group of all *p* values

^c^Elevated blood glucose ≥ 100 mg/dL after minimum 6-h fast

**Fig. 1:**
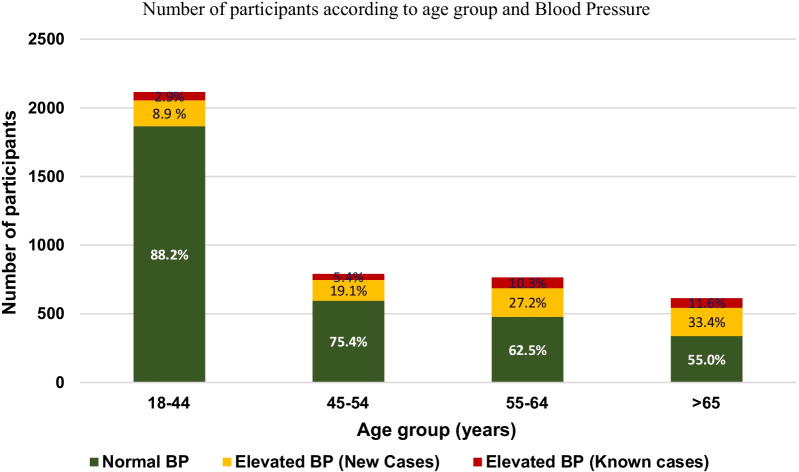
Number of participants according to age group and blood pressure

Of the 4284 individuals in the sampled cohort, 21.2% (n = 910) had elevated BP. Also 158 of 910 (17.4%) with elevated BP had a prior diagnosis of HTN and represent 62.2% of all individuals with a prior diagnosis of HTN (158/254). 752 of 910 (82.6%) individuals detected with elevated BP were without a prior diagnosis of HTN and represent 18.7% of all participants without history of HTN. A separate analysis which included known cases with controlled hypertension showed a normal BP with mean systolic blood pressure (SBP) of 127 ± 19 mmHg, and mean diastolic pressure (DBP) of 77 ± 11 mmHg (Additional file 1). The proportion of new discovery of elevated BP progressively increased with advancing age: 19.1% in 45–54 year old, 27.2% in 55–64 year old, and 33.4% among those with age > 65 years (Fig. 1).

Gender was similar between Normal BP and Elevated BP categories (Table 1). Age, weight, body mass index (BMI), and waist circumference were all higher in the Elevated BP categories compared with the Normal BP (all *p* < 0.001). Fasting blood glucose concentration was significantly higher in the all elevated BP and Elevated BP among Non-Known Hypertensive subjects. The prevalence of smoking history, alcohol consumption, and elevated blood glucose are all higher in the Elevated BP categories compared with the Normal BP category (all *p* < 0.005).

**Fig. 2:**
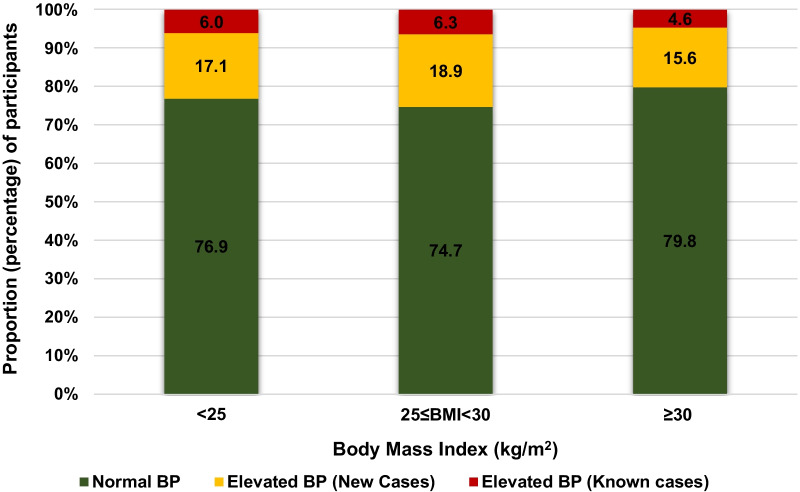
Number of participants by BMI and BP class

**Fig. 3:**
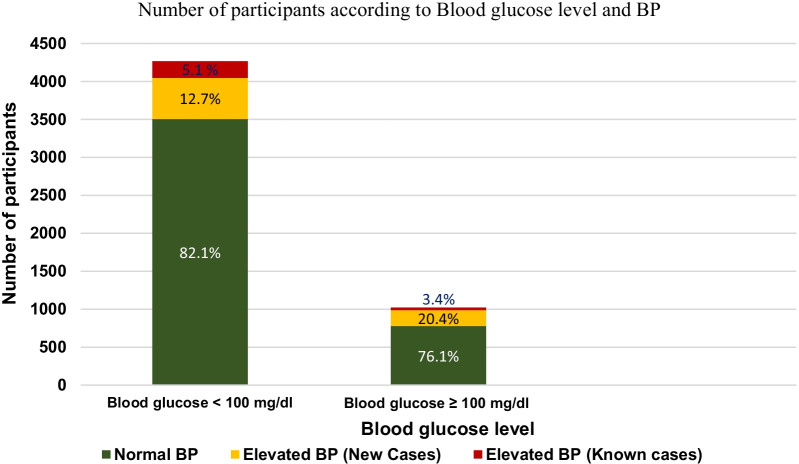
Number of participants according to blood glucose level and BP

The distribution of BP category was similar across BMI categories (Fig. 2). An elevated BG was found in 18.2% (n = 779) of participants, with a greater proportion of elevated BP among those participants with BG ≥ 100 mg/dL (Fig. 3). Among the 713 participants (16.6%) with a reported diagnosis of diabetes, 616 (79.1%) had a BG ≥ 100 mg/dL; BG was elevated in 163 participants without a reported diagnosis of diabetes (3.8% of the total screened population, 4.6% among participants without a reported diagnosis of diabetes) (Additional file 1).

### Independent risk factors of elevated BP in the 3 groups: general screened population, known hypertensive population and the newly discovered elevated BP sub-groups

Table 2 presents a multivariate analysis results of risk factors in the Normal BP group versus each of the three Elevated BP categories: All Elevated BP, Elevated BP in Known Hypertensive subjects, and Elevated BP in Non-Known Hypertensive subjects. The findings revealed that advanced age was independently associated with progressively increased risk of having elevated BP in all three comparisons (all *p* < 0.001). The relationship between Elevated BP category with overweight (25 ≤ BMI < 30 kg/m^2^) and obesity (BMI ≥ 30 kg/m^2^), weight, waist circumference, and fasting blood glucose were less consistent. Current smoking, alcohol consumption, and fasting blood glucose are significant predictors of Elevated BP in multivariate models (Table 2).

**Table 2:** Participants characteristics associated elevated BP in multivariate analysis

	Normal BP versus all elevated BP^a^		Normal BP versus elevated BP in known hypertensive subjects		Normal BP versus elevated BP in non-known hypertensive subjects	
	OR (95%CI)	*p* value	OR (95% CI)	*p* value	OR (95% CI)	*p* value
Age (years)						
18–44	Ref^d^		Ref^d^		Ref^d^	
45–54	1.8X [1.46–2.36]	< 0.001*	1.87 [1.10–3.18]	0.021*	1.76 [1.35–2.88]	< 0.001*
55–64	3.05 [2.41–3.85]	< 0.001*	3.81 [2.35–6.19]	< 0.001*	2.81 [2.17–3.63]	< 0.001*
≥ 65	3.91 [3.03–5.03]	< 0.001*	4.43 [2.62–7.49]	< 0.001*	3.63 [2.75–4.78]	< 0.001*
Weight (Kg)	1.02 [1.00–1.04]	0.024*	1.02 [0.99–1.06]	0.187	1.03 [1.01–1.05]	0.009*
BMI (kg/m^2^)						
< 25 kg/m^2^	Ref^d^		Ref^d^		Ref^d^	
25 ≤ BMI < 30 kg/m^2^	0.55 [0.44–0.71]	< 0.001*	0.80 [0.49–1.30]	0.368	0.49 [0.38–0.64]	< 0.001*
≥ 30 kg/m^2^	0.73 [0.48–1.10]	0.134	0.93 [0.42–2.11]	0.875	0.63 [0.40–1.00]	< 0.050*
Waist circumference (Cm)	1.01 [1.00–1.02]	< 0.001*	1.01 [1.00–1.02]	0.012*	1.01 [1.00–1.02]	0.001*
Smoking history (%)						
Never smoked	Ref^d^		Ref^d^		Ref^d^	
Ex-smoker	1.04 [0.83–1.30]	0.753	1.35 [0.86–2.14]	0.189	1.09 [0.85–1.39]	0.493
Current smoker	5.06 [4.05–6.31]	< 0.001*	5.71 [3.68–8.86]	< 0.001*	5.7X [4.50–7.25]	< 0.001*
Alcohol history (%)						
Never	Ref^d^		Ref^d^		Ref^d^	
0–5 years	2.48 [1.85–3.32]	< 0.001*	2.50 [1.37–4.56]	0.003*	2.48 [1.80–3.51]	< 0.001*
6–10 years	2.76 [1.85–3.32]	< 0.001*	3.38 [1.72–6.28]	< 0.001*	2.73 [1.90–3.94]	< 0.001*
11–15 years	2.79 [1.92–4.06]	< 0.001*	3.21 [1.55–6.61]	0.002*	3.04 [2.03–4.54]	< 0.001*
> 15 years	1.97 [1.57–2.48]	< 0.001*	1.52 [0.93–2.47]	0.092*	2.11 [1.64–2.71]	< 0.001*
Fasting blood glucose (mg/dL)	1.00 [1.00–1.01]	0.034*	1.01 [1.00–1.02]	0.007	1.00 [1.00–1.01]	0.055
Elevated blood glucose (%)^b^	0.91 [0.73–1.14]	0.426	0.16 [0.08–0.33]	< 0.001	1.15 [0.91–1.46]	0.235

^a^Elevated Blood pressure was defined as a systolic blood pressure (SBP) ≥ 140 mmHg and/or diastolic blood pressure (DBP) ≥ 90 mmHg

^b^Comparison by unpaired t-test or χ^2^ versus Normal BP

^c^Elevated blood glucose ≥ 100 mg/dL after minimum 6-h fast

^d^Ref: reference category

Table shows results from the multivariate analysis assessing independent participants’ characteristics associated to the elevated BP, comparison between each time the normal BP group versus elevated BP, versus poorly controlled BP and versus new discovered cases BP groups

## Discussion

This report revealed two main unexpected findings: A high rate of Elevated BP among individuals not known to have hypertension (18.7%), a higher rate of elevated BP in individuals with a known diagnosis of hypertension (62.2%), and high rates of elevated blood glucose (79.1%). High rates of elevated BP and elevated BG identified through a health screening campaign in this Rwandan district were surprising given the rural characteristics of the district and relatively low population age (46.4 ± 15.8 years).

### Awareness and hypertension control in this study

This report identified a high prevalence of elevated BP in individuals not previously known to have hypertension. This finding confirms both a high prevalence and a poor awareness of HTN typical of [17] especially in Sub-Sahara Africa [6, 22]. However, it is lower compared to the pooled weighted awareness rate of 16.9% in 1990, 29.2% in 2000 and 33.7% in 2010 reported by Adeloye et al. [23] from population-based studies on hypertension in Africa. The availability of data on awareness rates of hypertension in sub-Saharan Africa (SSA) in general, and from rural areas in particular, are scattered and generated by a wide range of studies differing in methodology thus limiting the opportunity for reliable comparisons [7, 24, 25]. However, the recent study by Chow et al. [26] reports that only one in three individual is aware of their hypertension status (i.e. higher than in our study), and about only 8% have their blood pressure controlled (i.e. lower than in our study). The poor treatment control in African settings has been largely reported by other authors, however the 37.8% reported rate by this study is higher than the reported average of 23.4% [26]. The high proportion of rural dwellers with uncontrolled hypertension in our study can be partially explained by geographically inaccessibility to health facilities for treatment monitoring.

### High prevalence found in this study

This study identified an overall prevalence of Elevated BP of 21.2% (95% CI = 20.0–22.4%). A prior population-based study in Rwanda estimated the prevalence of HTN in people between 15 and 64 years old at 15.3% (16.4% for males and 14.4% for females) [22], indicating that ~ 1 million people are living with HTN in Rwanda. The reported rate of elevated BP in this study was also lower than the 27.7% reported by a workplace-screening program conducted in an urban Ethiopian setting [27]. Other studies from rural areas of Sub-Saharan Africa have reported variable rates of HTN prevalence that ranges from 5 to 52% [8, 28, 29]. Adeloye et al. [23] had estimated hypertension pooled prevalence pooled in Africa at 26.1% (95% CI: 23.6–33.6). In line with those other pooled data, the results presented in the current study confirm a serious concern of the rising prevalence of hypertension in rural Rwanda. This threat is further exacerbated by an under-resourced healthcare system; the nationwide network of healthcare clinics available to treat NCDs in Rwanda only serve approximately 80,000 patients, representing a coverage of < 10% [30]. These data indicate that the majority of Rwandan adults with HTN are not only untreated, but undiagnosed.

### Participants’ characteristics in this study

The participant characteristics associated with elevated BP in this study are similar to the preliminary data reported by Muggli et al. [28] from another screening event held in the rural area of the District of Nyaruguru (Southern Rwanda) which found a much low prevalence of hypertension at 8.8%. However, that screening event reported a median age of 32 years, much younger than 46 ± 16 years reported in this study. This lower age likely contributes to the low prevalence of hypertension found in their report. As reported in other studies [7, 10, 17, 29], gender was not significantly associated to elevated BP in our study. However, consistent with other studies [25, 31, 32], advancing age was a significant predictor of elevated BP in this study.

The association between overweight and elevated blood pressure has been long-established, including in African populations [15, 33–35]. This study revealed a high prevalence of overweight and obesity as noted in other prior studies conducted in rural Rwandan areas [22]. The relationship between BP and anthropometric indices such as BMI, WC, waist to hip ratio (WHpR), and waist to height ratio (WHtR) have been described in others studies [33]. Our study was not powered to assess this comparison between overweight indices, and some indices were not available for analysis. Therefore, we added all raw data on weight, height, BMI and WC into analysis both univariate and multivariate after assess collinearity with correlation matrix between variables. As the distribution of BP category was similar across BMI categories, it was not conclusive to clearly draw a conclusion on the association between the BMI categories and the elevated BP in our study. This was probably due to the small sample size in cells.

Nevertheless, this finding is unexpected and further work is needed to validate the association identified in this study since these are in contrast with other reports from rural India [34] and Nigerian [35] which identify a higher BMI as a major risk factor for hypertension [18, 36, 37].

Other risk factors identified in multivariable analysis as independently associated to elevated BP are in line with previous Rwanda WHO STEPs study by Nahimana et al. [22]. In their nationwide study, a logistic regression model revealed that age, alcohol consumption, blood glucose levels, and raised BMI were significantly associated with hypertension, a finding confirmed in this study.

### Implication for strategy for the prevention, diagnosis and treatment of hypertension in Rwanda

This study brings additional evidence to support tailored measures for the prevention, diagnosis and treatment of hypertension in rural populations in Rwanda. Recent reviews analyzing root causes for poor blood pressure control in Rwanda [38] and in Eastern Sub-Saharan Africa [39] highlight this unmet healthcare need. On a global scale, the World Health Organization (WHO) has created a target to reduce heart attacks and stroke by 25% by 2025 [17], and the World Heart Federation (WHF) launched a roadmap focusing on raised BP awareness, treatment, and control during the 2015 World Health Assembly in Geneva [40]. Monitoring through systematically organized periodic screening campaigns for HTN, diabetes, and other NCD risk factors in rural population remains a key strategy for optimizing treatment and control [24]. Early detection with opportunistic screening campaigns can also mitigate multiple barriers like poor health education literacy [38] and low socio-economic status [39]. However, given the need to balance competing healthcare priorities including infectious diseases, nutritional deficiencies, and maternal and perinatal morbidity and mortality, a reallocation of healthcare resources towards continuous monitoring of NCDs in LMICs is necessary [6].

### Study limitations

This study fills in a scientific data gap and its large sample size is adequate for subgroup comparisons. This is also one of the first studies to characterize the blood pressure and blood glucose profiles in a remote rural area in Rwanda through a community screening. However, it has also some limitations. First, given the current study is not a systematic population-based screening, bias from participant self-selection likely influenced the results of this study. Second, given the cross-sectional design of this screening study, we are unable to determine temporality and causality in the study; therefore, causation can only be inferred. Third, like other opportunistic screening studies wherein the BP measurement are taken at a single visit, this is indeed a limitation. However, the BP measurements were taken through a rigorous and standardized protocol (3 times, with 1-min intervals, after rest period). Additionally, all subjects with elevated BP were referred to nearest health facilities -as usual procedure, where hypertension diagnosis was confirmed for all the detected elevated BP cases, and followed an appropriate management. The follow up data are not part of this manuscript. The study reports only elevated BP and not prevalence of hypertension because the three blood pressure measurements were performed only on one occasion. Fourth, we were unable to explore the contribution of other risk factors such as unhealthy diet, salt intake, physical activity and psychosocial stress, the lack of information on those risk factors that significantly affects the prevalence of hypertension and limits the strength of the conclusions. Such parameters were not easy to legitimate and scientifically evaluate through an opportunistic screening campaign, so the related information were not available during casual data collection.” Additionally, the quantification of alcohol intake and smoking where rather empirical in the region where traditional alcoholic drinks [41] and traditional pipe smoking were hard to define according to international standards [42]. There might be also social desirability biases to underestimating some of the lifestyle and behavioral questions, such as smoking and alcohol consumptions. Despite these limitations, the study makes significant contribution and fills a substantial gap in the current Rwanda and regional context. The use of standardized and robust methodologies, tools and high response rate observed in the study increased its representativeness and strengthens its value in informing the CVDs prevention by enabling tailored preventive measures and optimize the treatment control.

## Conclusion

This opportunistic screening findings confirm a high rate of newly discovered elevated BP and poor control among rural population in Rwanda. This study identified age, current smoking, and alcohol history and waist circumference to be the risks associated with elevated BP. Having such high rate of newly discovered abnormal BP from that rural population would imply an epidemiological risk profile transition for hypertension, that needs further studies. Nevertheless, these data support the need to strengthen also in rural areas of Rwanda an adequate strategy for the prevention, early diagnosis and treatment of hypertension. Future longitudinal studies to analyse in more details the specific CVDs risk in that population are needed.

## Supplementary Information

**Additional file 1.** Additional descriptive analysis on participants’ characteristics and diabetes cases (old versus new cases).

## Data Availability

The dataset used and/or analyzed during this study is available from the corresponding author on reasonable request.

## Abbreviations

BP: Blood pressure
BG: Blood glucose
BMI: Body Mass Index
CVDs: Cardiovascular diseases
DBP: Diastolic blood pressure
HC: Health center
HBC: Health belief models
HTN: Hypertension
LMICs: Low middle income countries
NCDs: Non-communicable diseases center
NISR: National Institute of Statistics of Rwanda
RBC: Rwanda Biomedical Center
SBP: Systolic blood pressure
WC: Waist circumference
WHpR: Waist to hip ratio
WHtR: Waist to height ratio
WHO: World Health Organization
WHF: World Heart Federation
