# Supplementary material for: High rates of undiagnosed and uncontrolled hypertension upon a screening campaign in rural Rwanda: a cross-sectional study
Source: BMC Cardiovasc Disord. 2022 Apr 26;22:197. doi: 10.1186/s12872-022-02606-9 (PMC9044706; doi:10.1186/s12872-022-02606-9)
Supplement: Supplementary file 1 — Additional file 1. Additional descriptive analysis on participants’ characteristics and diabetes cases (old versus new cases). [file 12872_2022_2606_MOESM1_ESM.docx]

**High Rates of Undiagnosed and Uncontrolled Hypertension Upon a Screening Campaign in Rural Rwanda: A Cross-Sectional Study**

Evariste Ntaganda^1^,

Regine Mugeni^2^_,_

Emmanuel Harerimana^2^,

Gedeon Ngoga^3^,

Symaque Dusabeyezu^3^,

Francois Uwinkindi^2^,

Jean Nepo A. Utumatwishima^2^,

Eugene Mutimura^4^,

Victor G. Davila-Roman^5^,

Kenneth Schechtman^6^,

Aurore Nishimwe^7^

Laurence Twizeyimana^7^

Angela L. Brown^5^ ;

W. Todd Cade^8^

Marcus Bushaku^7^

Lisa De Las Fuentes^5^

Dominic Reeds^5^

Marc Twagirumukiza^10^

1. Rwanda Biomedical Center (RBC), Rwanda Ministry of Health, Kigali, Rwanda.
2. Rwamagana Provincial Hospital, Rwamagana, Eastern Province, Rwanda
3. Partners in Health (PIH)/Inshuti Mu Buzima, Rwinkwavu, Rwanda
4. National Council for Science and Technology (NCST), Kigali, Rwanda
5. Cardiovascular Division, Department of Medicine, Washington University in St. Louis, St Louis, Missouri, USA
6. Division of Biostatistics, Washington University in St. Louis, St. Louis, Missouri, USA
7. Regional Alliance for Sustainable Development (RASD Rwanda)
8. Duke University School of Medicine, 27710, Durham, NC, USA
9. School of Medicine and Pharmacy, College of Medicine and Health Sciences, University of Rwanda
10. Faculty of Medicine and Health Sciences, Ghent University, Ghent, Belgium

**Corresponding Author:**

Regine Mugeni, MD

Clinical Scientist

Rwamagana Regional Hospital

Rwamagana, Rwanda

E-mail: [pacisreg@gmail.com](mailto:pacisreg@gmail.com)

Tel: +250 788 552 426

**Supplementary materials**

**Table S1. Descriptive Statistics**

|  | N | Range | Minimum | Maximum | Mean | Std. Deviation |
| --- | --- | --- | --- | --- | --- | --- |
| Age | 4284 | 80 | 18 | 98 | 46,43 | 15,806 |
| Height (m) | 4284 | ,67 | 1,13 | 1,80 | 1,6630 | ,04101 |
| Weight (Kg) | 4284 | 58,00 | 48,00 | 106,00 | 73,5786 | 9,26374 |
| Waist circumference (Centimeters) | 4284 | 82,0 | 30,0 | 112,0 | 74,386 | 16,5828 |
| Systolic BP (mmHg) | 4284 | 121 | 75 | 196 | 126,82 | 18,579 |
| Diastolic BP (mmHg) | 4284 | 50 | 49 | 99 | 75,61 | 10,759 |
| Blood Glucose/ mg/dl | 4284 | 206 | 67 | 273 | 100,87 | 21,654 |
| Body Mass Index (kg/m²) | 4284 | 21,6 | 16,8 | 38,4 | 26,659 | 3,6761 |
| Valid N (listwise) | 4284 |  |  |  |  |  |

**Table S2. Gender**

|  | | Frequency | Percent | Valid Percent | Cumulative Percent |
| --- | --- | --- | --- | --- | --- |
| Valid | Female | 2417 | 56,4 | 56,4 | 56,4 |
|  | Male | 1867 | 43,6 | 43,6 | 100,0 |
|  | Total | 4284 | 100,0 | 100,0 |  |

| **Table S3. Participants district of provenance** | | | | | |
| --- | --- | --- | --- | --- | --- |
|  | | Frequency | Percent | Valid Percent | Cumulative Percent |
| Valid | Kayonza | 46 | 1,1 | 1,1 | 1,1 |
|  | Kirehe | 4183 | 97,6 | 97,6 | 98,7 |
|  | Ngoma | 55 | 1,3 | 1,3 | 100,0 |
|  | Total | 4284 | 100,0 | 100,0 |  |

**Table S4. Participants sector of provenance**

|  | | Frequency | Percent | Valid Percent | Cumulative Percent |
| --- | --- | --- | --- | --- | --- |
| Valid | Gahara | 923 | 21,5 | 21,5 | 21,5 |
|  | Gatore | 552 | 12,9 | 12,9 | 34,4 |
|  | Kigarama | 664 | 15,5 | 15,5 | 49,9 |
|  | Kigina | 885 | 20,7 | 20,7 | 70,6 |
|  | Kirehe | 312 | 7,3 | 7,3 | 77,9 |
|  | Nasho | 891 | 20,8 | 20,8 | 98,7 |
|  | Others | 57 | 1,3 | 1,3 | 100,0 |
|  | Total | 4284 | 100,0 | 100,0 |  |

**Table S5. Old or New HTN case**

|  | | Frequency | Percent | Valid Percent | Cumulative Percent |
| --- | --- | --- | --- | --- | --- |
| Valid | Known case | 254 | 5,9 | 25,2 | 25,2 |
|  | New case | 752 | 17,6 | 74,8 | 100,0 |
|  | Total | 1006 | 23,5 | 100,0 |  |
| Missing | System | 3278 | 76,5 |  |  |
| Total | | 4284 | 100,0 |  |  |

**Table S6. Last food intake**

|  | | Frequency | Percent | Valid Percent | Cumulative Percent |
| --- | --- | --- | --- | --- | --- |
| Valid | ≥ 6 hours | 2494 | 58,2 | 58,2 | 58,2 |
|  | 3-6 hours | 1164 | 27,2 | 27,2 | 85,4 |
|  | 1-3Hrs | 614 | 14,3 | 14,3 | 99,7 |
|  | < 1 hour | 12 | ,3 | ,3 | 100,0 |
|  | Total | 4284 | 100,0 | 100,0 |  |

**Table S7. Old or New Diabetes case**

|  | | Frequency | Percent | Valid Percent | Cumulative Percent |
| --- | --- | --- | --- | --- | --- |
| Valid | Known case | 713 | 16,6 | 81,4 | 81,4 |
|  | New case | 163 | 3,8 | 18,6 | 100,0 |
|  | Total | 876 | 20,4 | 100,0 |  |
| Missing | System | 3408 | 79,6 |  |  |
| Total | | 4284 | 100,0 |  |  |

**Table S8. Smoking**

|  | | Frequency | Percent | Valid Percent | Cumulative Percent |
| --- | --- | --- | --- | --- | --- |
| Valid | Never smoke | 2827 | 66,0 | 66,0 | 66,0 |
|  | Ex-smoker | 821 | 19,2 | 19,2 | 85,2 |
|  | Current smoker | 636 | 14,8 | 14,8 | 100,0 |
|  | Total | 4284 | 100,0 | 100,0 |  |

**Table S9. Smoking duration**

|  | | Frequency | Percent | Valid Percent | Cumulative Percent |
| --- | --- | --- | --- | --- | --- |
| Valid | NA | 3057 | 71,4 | 71,4 | 71,4 |
|  | 0-5 years | 238 | 5,6 | 5,6 | 76,9 |
|  | 6-10 years | 162 | 3,8 | 3,8 | 80,7 |
|  | 11-15 years | 58 | 1,4 | 1,4 | 82,0 |
|  | > 15 years | 769 | 18,0 | 18,0 | 100,0 |
|  | Total | 4284 | 100,0 | 100,0 |  |

**Table S10. Alcohol intake**

|  | | Frequency | Percent | Valid Percent | Cumulative Percent |
| --- | --- | --- | --- | --- | --- |
| Valid | Never drink | 1257 | 29,3 | 29,3 | 29,3 |
|  | Ex-drinker | 1262 | 29,5 | 29,5 | 58,8 |
|  | Drink occasionally | 1159 | 27,1 | 27,1 | 85,9 |
|  | Daily 1 drink for woman or 1-2 drinks for man | 195 | 4,6 | 4,6 | 90,4 |
|  | Daily 2 drinks for woman or 3 drinks for man | 123 | 2,9 | 2,9 | 93,3 |
|  | Daily 3 drinks for woman or 4 drinks for man | 33 | ,8 | ,8 | 94,0 |
|  | Daily > 3 drinks for woman or > 4 drinks for man | 255 | 6,0 | 6,0 | 100,0 |
|  | Total | 4284 | 100,0 | 100,0 |  |

**Table S11. Alcohol duration**

|  | | Frequency | Percent | Valid Percent | Cumulative Percent |
| --- | --- | --- | --- | --- | --- |
| Valid | NA | 1552 | 36,2 | 36,2 | 36,2 |
|  | 0-5 years | 537 | 12,5 | 12,5 | 48,8 |
|  | 6-10 years | 317 | 7,4 | 7,4 | 56,2 |
|  | 11-15 years | 234 | 5,5 | 5,5 | 61,6 |
|  | > 15 years | 1644 | 38,4 | 38,4 | 100,0 |
|  | Total | 4284 | 100,0 | 100,0 |  |

**Table S12. Elevated Blood Pressure (SBP >= 140 and/or DBP >= 90)**

|  | | Frequency | Percent | Valid Percent | Cumulative Percent |
| --- | --- | --- | --- | --- | --- |
| Valid | No | 3374 | 78,8 | 78,8 | 78,8 |
|  | Yes | 910 | 21,2 | 21,2 | 100,0 |
|  | Total | 4284 | 100,0 | 100,0 |  |

**Table S13. Elevated Blood glucose**

|  | | Frequency | Percent | Valid Percent | Cumulative Percent |
| --- | --- | --- | --- | --- | --- |
| Valid | No | 3505 | 81,8 | 81,8 | 81,8 |
|  | Yes | 779 | 18,2 | 18,2 | 100,0 |
|  | Total | 4284 | 100,0 | 100,0 |  |

**Table S14. Aged 45 years or more**

|  | | Frequency | Percent | Valid Percent | Cumulative Percent |
| --- | --- | --- | --- | --- | --- |
| Valid | Aged of less than 45 years | 2115 | 49,4 | 49,4 | 49,4 |
|  | Aged of 45 years or more | 2169 | 50,6 | 50,6 | 100,0 |
|  | Total | 4284 | 100,0 | 100,0 |  |

**Table S15. Alcohol History**

|  | | Frequency | Percent | Valid Percent | Cumulative Percent |
| --- | --- | --- | --- | --- | --- |
| Valid | Never drinks | 1257 | 29,3 | 29,3 | 29,3 |
|  | Ex-drinker | 1262 | 29,5 | 29,5 | 58,8 |
|  | Current drinker | 1765 | 41,2 | 41,2 | 100,0 |
|  | Total | 4284 | 100,0 | 100,0 |  |

**Continuous variables means per subjects characteristics**

**Table S16. Means per gender**

| Gender | | Waist circumference (Centimeters) | Body Mass Index (kg/m²) | Blood Glucose/ mg/dl | Systolic BP (mmHg) | Diastolic BP (mmHg) |
| --- | --- | --- | --- | --- | --- | --- |
| Female | Mean | 76,373 | 27,840 | 101,16 | 125,89 | 75,46 |
|  | Std. Deviation | 15,6675 | 4,0342 | 21,958 | 18,891 | 10,681 |
| Male | Mean | 71,813 | 25,131 | 100,51 | 128,01 | 75,81 |
|  | Std. Deviation | 17,3672 | 2,4090 | 21,255 | 18,104 | 10,858 |
| Total | Mean | 74,386 | 26,659 | 100,87 | 126,82 | 75,61 |
|  | Std. Deviation | 16,5828 | 3,6761 | 21,654 | 18,579 | 10,759 |

**Table S17. Waist circumference (Centimeters) Body Mass Index (kg/m²) Blood Glucose/ mg/dl Systolic BP (mmHg) Diastolic BP (mmHg) per Old or New HTN case**

| Old or New HTN case | | Waist circumference (Centimeters) | Body Mass Index (kg/m²) | Blood Glucose/ mg/dl | Systolic BP (mmHg) | Diastolic BP (mmHg) |
| --- | --- | --- | --- | --- | --- | --- |
| Known case | Mean | 77,504 | 26,939 | 105,11 | 145,44 | 84,08 |
|  | Std. Deviation | 17,3983 | 4,1894 | 30,209 | 16,895 | 10,333 |
| New case | Mean | 80,517 | 27,726 | 105,03 | 153,83 | 88,25 |
|  | Std. Deviation | 18,6315 | 4,6978 | 23,669 | 13,138 | 9,082 |
| Total | Mean | 79,756 | 27,527 | 105,05 | 151,72 | 87,20 |
|  | Std. Deviation | 18,3662 | 4,5855 | 25,463 | 14,633 | 9,581 |

**Table S18. Waist circumference (Centimeters) Body Mass Index (kg/m²) Blood Glucose/ mg/dl Systolic BP (mmHg) Diastolic BP (mmHg) per Old or New Diabetes case**

| Old or New Diabetes case | | Waist circumference (Centimeters) | Body Mass Index (kg/m²) | Blood Glucose/ mg/dl | Systolic BP (mmHg) | Diastolic BP (mmHg) |
| --- | --- | --- | --- | --- | --- | --- |
| Known case | Mean | 77,442 | 27,083 | 117,41 | 129,87 | 77,31 |
|  | Std. Deviation | 17,5104 | 4,2917 | 22,366 | 20,537 | 11,109 |
| New case | Mean | 76,736 | 27,309 | 117,38 | 127,35 | 76,10 |
|  | Std. Deviation | 17,8266 | 4,0553 | 24,486 | 18,153 | 10,703 |
| Total | Mean | 77,311 | 27,125 | 117,40 | 129,40 | 77,08 |
|  | Std. Deviation | 17,5616 | 4,2474 | 22,761 | 20,129 | 11,038 |

**Table S19. Waist circumference (Centimeters) Body Mass Index (kg/m²) Blood Glucose/ mg/dl Systolic BP (mmHg) Diastolic BP (mmHg) * Smoking**

| Smoking | | Waist circumference (Centimeters) | Body Mass Index (kg/m²) | Blood Glucose/ mg/dl | Systolic BP (mmHg) |
| --- | --- | --- | --- | --- | --- |
| Never smoke | Mean | 72,461 | 26,314 | 99,69 | 123,56 |
|  | Std. Deviation | 16,4995 | 3,3598 | 20,700 | 16,160 |
| Ex-smoker | Mean | 74,532 | 26,322 | 100,18 | 127,92 |
|  | Std. Deviation | 14,4510 | 3,3788 | 19,855 | 19,167 |
| Current smoker | Mean | 82,756 | 28,627 | 107,05 | 139,87 |
|  | Std. Deviation | 16,9366 | 4,6492 | 26,488 | 21,733 |
| Total | Mean | 74,386 | 26,659 | 100,87 | 126,82 |
|  | Std. Deviation | 16,5828 | 3,6761 | 21,654 | 18,579 |

**Table S20. Waist circumference (Centimeters) Body Mass Index (kg/m²) Blood Glucose/ mg/dl Systolic BP (mmHg) Diastolic BP (mmHg) * Smoking**

| Smoking | | Diastolic BP (mmHg) |
| --- | --- | --- |
| Never smoke | Mean | 74,07 |
|  | Std. Deviation | 9,941 |
| Ex-smoker | Mean | 76,09 |
|  | Std. Deviation | 10,807 |
| Current smoker | Mean | 81,85 |
|  | Std. Deviation | 11,842 |
| Total | Mean | 75,61 |
|  | Std. Deviation | 10,759 |

**Table S21. Waist circumference (Centimeters) Body Mass Index (kg/m²) Blood Glucose/ mg/dl Systolic BP (mmHg) Diastolic BP (mmHg) * Alcohol intake**

| Alcohol intake | | Waist circumference (Centimeters) | Body Mass Index (kg/m²) | Blood Glucose/ mg/dl |
| --- | --- | --- | --- | --- |
| Never drink | Mean | 71,496 | 26,616 | 100,59 |
|  | Std. Deviation | 16,8517 | 3,4077 | 21,317 |
| Ex-drinker | Mean | 74,667 | 26,364 | 98,83 |
|  | Std. Deviation | 15,2662 | 3,3994 | 19,831 |
| Drink occasionally | Mean | 75,643 | 26,779 | 101,02 |
|  | Std. Deviation | 16,5999 | 3,8499 | 22,446 |
| Daily 1 drink for woman or 1-2 drinks for man | Mean | 72,662 | 25,819 | 104,32 |
|  | Std. Deviation | 15,2125 | 3,2876 | 23,576 |
| Daily 2 drinks for woman or 3 drinks for man | Mean | 72,215 | 25,660 | 100,15 |
|  | Std. Deviation | 15,5270 | 3,0728 | 16,037 |
| Daily 3 drinks for woman or 4 drinks for man | Mean | 73,394 | 24,190 | 101,33 |
|  | Std. Deviation | 16,2287 | 1,9400 | 18,874 |
| Daily > 3 drinks for woman or > 4 drinks for man | Mean | 84,022 | 29,234 | 109,36 |
|  | Std. Deviation | 18,6226 | 4,8227 | 26,733 |
| Total | Mean | 74,386 | 26,659 | 100,87 |
|  | Std. Deviation | 16,5828 | 3,6761 | 21,654 |

**Table S22. Waist circumference (Centimeters) Body Mass Index (kg/m²) Blood Glucose/ mg/dl Systolic BP (mmHg) Diastolic BP (mmHg) * Alcohol intake**

| Alcohol intake | | Systolic BP (mmHg) | Diastolic BP (mmHg) |
| --- | --- | --- | --- |
| Never drink | Mean | 122,15 | 73,43 |
|  | Std. Deviation | 15,925 | 9,942 |
| Ex-drinker | Mean | 125,35 | 74,75 |
|  | Std. Deviation | 18,189 | 10,292 |
| Drink occasionally | Mean | 129,54 | 77,13 |
|  | Std. Deviation | 19,432 | 11,070 |
| Daily 1 drink for woman or 1-2 drinks for man | Mean | 120,72 | 71,99 |
|  | Std. Deviation | 11,464 | 8,098 |
| Daily 2 drinks for woman or 3 drinks for man | Mean | 126,36 | 76,05 |
|  | Std. Deviation | 13,164 | 9,473 |
| Daily 3 drinks for woman or 4 drinks for man | Mean | 131,03 | 78,85 |
|  | Std. Deviation | 16,185 | 11,353 |
| Daily > 3 drinks for woman or > 4 drinks for man | Mean | 149,06 | 85,90 |
|  | Std. Deviation | 17,497 | 10,659 |
| Total | Mean | 126,82 | 75,61 |
|  | Std. Deviation | 18,579 | 10,759 |

**Table S23. Participants characteristics by Gender**

**Table S23a. Report**

| Gender | | Age | Body Mass Index (kg/m²) | Systolic BP (mmHg) | Diastolic BP (mmHg) |
| --- | --- | --- | --- | --- | --- |
| Female | Mean | 45,69 | 27,840 | 125,89 | 75,46 |
|  | Std. Deviation | 15,860 | 4,0342 | 18,891 | 10,681 |
| Male | Mean | 47,39 | 25,131 | 128,01 | 75,81 |
|  | Std. Deviation | 15,689 | 2,4090 | 18,104 | 10,858 |
| Total | Mean | 46,43 | 26,659 | 126,82 | 75,61 |
|  | Std. Deviation | 15,806 | 3,6761 | 18,579 | 10,759 |

**Table S23b. ANOVA Table**

|  | | | Sum of Squares | df |
| --- | --- | --- | --- | --- |
| Age * Gender | Between Groups | (Combined) | 3018,732 | 1 |
|  | Within Groups | | 1067046,229 | 4282 |
|  | Total | | 1070064,961 | 4283 |
| Body Mass Index (kg/m²) * Gender | Between Groups | (Combined) | 7730,221 | 1 |
|  | Within Groups | | 50148,249 | 4282 |
|  | Total | | 57878,470 | 4283 |
| Systolic BP (mmHg) * Gender | Between Groups | (Combined) | 4720,420 | 1 |
|  | Within Groups | | 1473745,267 | 4282 |
|  | Total | | 1478465,687 | 4283 |
| Diastolic BP (mmHg) * Gender | Between Groups | (Combined) | 126,748 | 1 |
|  | Within Groups | | 495642,435 | 4282 |
|  | Total | | 495769,183 | 4283 |

**Table S23c. ANOVA Table**

|  | | | Mean Square | F | Sig. |
| --- | --- | --- | --- | --- | --- |
| Age * Gender | Between Groups | (Combined) | 3018,732 | 12,114 | <,001 |
|  | Within Groups | | 249,193 |  |  |
|  | Total | |  |  |  |
| Body Mass Index (kg/m²) * Gender | Between Groups | (Combined) | 7730,221 | 660,059 | <,001 |
|  | Within Groups | | 11,711 |  |  |
|  | Total | |  |  |  |
| Systolic BP (mmHg) * Gender | Between Groups | (Combined) | 4720,420 | 13,715 | <,001 |
|  | Within Groups | | 344,172 |  |  |
|  | Total | |  |  |  |
| Diastolic BP (mmHg) * Gender | Between Groups | (Combined) | 126,748 | 1,095 | ,295 |
|  | Within Groups | | 115,750 |  |  |
|  | Total | |  |  |  |

**Table S24. Participants characteristics by gender, stratified by Aged 45 years or more**

**Table S24a. Report**

| Aged 45 years or more | Gender | | Body Mass Index (kg/m²) | Systolic BP (mmHg) | Diastolic BP (mmHg) |
| --- | --- | --- | --- | --- | --- |
| Aged of less than 45 years | Female | Mean | 27,536 | 120,05 | 72,71 |
|  |  | N | 1221 | 1221 | 1221 |
|  |  | Std. Deviation | 3,9760 | 14,699 | 9,678 |
|  | Male | Mean | 24,972 | 123,41 | 73,35 |
|  |  | N | 894 | 894 | 894 |
|  |  | Std. Deviation | 2,2861 | 15,275 | 10,236 |
|  | Total | Mean | 26,452 | 121,47 | 72,98 |
|  |  | N | 2115 | 2115 | 2115 |
|  |  | Std. Deviation | 3,5966 | 15,033 | 9,920 |
| Aged of 45 years or more | Female | Mean | 28,150 | 131,86 | 78,27 |
|  |  | N | 1196 | 1196 | 1196 |
|  |  | Std. Deviation | 4,0710 | 20,748 | 10,929 |
|  | Male | Mean | 25,277 | 132,24 | 78,07 |
|  |  | N | 973 | 973 | 973 |
|  |  | Std. Deviation | 2,5091 | 19,429 | 10,926 |
|  | Total | Mean | 26,861 | 132,03 | 78,18 |
|  |  | N | 2169 | 2169 | 2169 |
|  |  | Std. Deviation | 3,7417 | 20,163 | 10,926 |
| Total | Female | Mean | 27,840 | 125,89 | 75,46 |
|  |  | N | 2417 | 2417 | 2417 |
|  |  | Std. Deviation | 4,0342 | 18,891 | 10,681 |
|  | Male | Mean | 25,131 | 128,01 | 75,81 |
|  |  | N | 1867 | 1867 | 1867 |
|  |  | Std. Deviation | 2,4090 | 18,104 | 10,858 |
|  | Total | Mean | 26,659 | 126,82 | 75,61 |
|  |  | N | 4284 | 4284 | 4284 |
|  |  | Std. Deviation | 3,6761 | 18,579 | 10,759 |

**Table S24b. ANOVA Table**

|  | | | Sum of Squares | df |
| --- | --- | --- | --- | --- |
| Body Mass Index (kg/m²) * Aged 45 years or more | Between Groups | (Combined) | 179,020 | 1 |
|  | Within Groups | | 57699,450 | 4282 |
|  | Total | | 57878,470 | 4283 |
| Systolic BP (mmHg) * Aged 45 years or more | Between Groups | (Combined) | 119296,162 | 1 |
|  | Within Groups | | 1359169,525 | 4282 |
|  | Total | | 1478465,687 | 4283 |
| Diastolic BP (mmHg) * Aged 45 years or more | Between Groups | (Combined) | 28919,920 | 1 |
|  | Within Groups | | 466849,263 | 4282 |
|  | Total | | 495769,183 | 4283 |

**Table S24c. ANOVA Table**

|  | | | Mean Square | F | Sig. |
| --- | --- | --- | --- | --- | --- |
| Body Mass Index (kg/m²) * Aged 45 years or more | Between Groups | (Combined) | 179,020 | 13,285 | <,001 |
|  | Within Groups | | 13,475 |  |  |
|  | Total | |  |  |  |
| Systolic BP (mmHg) * Aged 45 years or more | Between Groups | (Combined) | 119296,162 | 375,837 | <,001 |
|  | Within Groups | | 317,415 |  |  |
|  | Total | |  |  |  |
| Diastolic BP (mmHg) * Aged 45 years or more | Between Groups | (Combined) | 28919,920 | 265,257 | <,001 |
|  | Within Groups | | 109,026 |  |  |
|  | Total | |  |  |  |

**Crosstabs

Table S25. Gender * Elevated Blood Pressure (SBP >= 140 and/or DBP >= 90)**

**Table S25a. Crosstab**

|  | | | Elevated Blood Pressure (SBP >= 140 and/or DBP >= 90) | | Total |
| --- | --- | --- | --- | --- | --- |
|  |  |  | No | Yes |  |
| Gender | Female | Count | 1922 | 495 | 2417 |
|  |  | % within Gender | 79,5% | 20,5% | 100,0% |
|  |  | % within Elevated Blood Pressure (SBP >= 140 and/or DBP >= 90) | 57,0% | 54,4% | 56,4% |
|  |  | % of Total | 44,9% | 11,6% | 56,4% |
|  | Male | Count | 1452 | 415 | 1867 |
|  |  | % within Gender | 77,8% | 22,2% | 100,0% |
|  |  | % within Elevated Blood Pressure (SBP >= 140 and/or DBP >= 90) | 43,0% | 45,6% | 43,6% |
|  |  | % of Total | 33,9% | 9,7% | 43,6% |
| Total | | Count | 3374 | 910 | 4284 |
|  |  | % within Gender | 78,8% | 21,2% | 100,0% |
|  |  | % within Elevated Blood Pressure (SBP >= 140 and/or DBP >= 90) | 100,0% | 100,0% | 100,0% |
|  |  | % of Total | 78,8% | 21,2% | 100,0% |

**Table S25b. Chi-Square Tests**

|  | Value | df | Asymptotic Significance (2-sided) | Exact Sig. (2-sided) | Exact Sig. (1-sided) |
| --- | --- | --- | --- | --- | --- |
| Pearson Chi-Square | 1,924^a^ | 1 | ,165 |  |  |
| Continuity Correction^b^ | 1,821 | 1 | ,177 |  |  |
| Likelihood Ratio | 1,920 | 1 | ,166 |  |  |
| Fisher's Exact Test |  |  |  | ,175 | ,089 |
| N of Valid Cases | 4284 |  |  |  |  |
| a. 0 cells (0,0%) have expected count less than 5. The minimum expected count is 396,58. | | | | | |
| b. Computed only for a 2x2 table | | | | | |

**Table S26. Gender * Elevated Blood glucose**

**Table S26a. Crosstab**

|  | | | Elevated Blood glucose | | Total |
| --- | --- | --- | --- | --- | --- |
|  |  |  | No | Yes |  |
| Gender | Female | Count | 1961 | 456 | 2417 |
|  |  | % within Gender | 81,1% | 18,9% | 100,0% |
|  |  | % within Elevated Blood glucose | 55,9% | 58,5% | 56,4% |
|  |  | % of Total | 45,8% | 10,6% | 56,4% |
|  | Male | Count | 1544 | 323 | 1867 |
|  |  | % within Gender | 82,7% | 17,3% | 100,0% |
|  |  | % within Elevated Blood glucose | 44,1% | 41,5% | 43,6% |
|  |  | % of Total | 36,0% | 7,5% | 43,6% |
| Total | | Count | 3505 | 779 | 4284 |
|  |  | % within Gender | 81,8% | 18,2% | 100,0% |
|  |  | % within Elevated Blood glucose | 100,0% | 100,0% | 100,0% |
|  |  | % of Total | 81,8% | 18,2% | 100,0% |

**Table S26b. Chi-Square Tests**

|  | Value | df | Asymptotic Significance (2-sided) | Exact Sig. (2-sided) | Exact Sig. (1-sided) |
| --- | --- | --- | --- | --- | --- |
| Pearson Chi-Square | 1,736^a^ | 1 | ,188 |  |  |
| Continuity Correction^b^ | 1,632 | 1 | ,201 |  |  |
| Likelihood Ratio | 1,741 | 1 | ,187 |  |  |
| Fisher's Exact Test |  |  |  | ,201 | ,101 |
| N of Valid Cases | 4284 |  |  |  |  |
| a. 0 cells (0,0%) have expected count less than 5. The minimum expected count is 339,49. | | | | | |
| b. Computed only for a 2x2 table | | | | | |

**Table S27. Elevated Blood Pressure (SBP >= 140 and/or DBP >= 90) * Old or New HTN case Crosstabulation**

**Table S27a. Count**

|  | | Old or New HTN case | | Total |
| --- | --- | --- | --- | --- |
|  |  | Known case | New case |  |
| Elevated Blood Pressure (SBP >= 140 and/or DBP >= 90) | No | 96 | 0 | 96 |
|  | Yes | 158 | 752 | 910 |
| Total | | 254 | 752 | 1006 |

**Table S27b. Chi-Square Tests**

|  | Value | df | Asymptotic Significance (2-sided) | Exact Sig. (2-sided) | Exact Sig. (1-sided) |
| --- | --- | --- | --- | --- | --- |
| Pearson Chi-Square | 314,204^a^ | 1 | <,001 |  |  |
| Continuity Correction^b^ | 309,841 | 1 | <,001 |  |  |
| Likelihood Ratio | 296,785 | 1 | <,001 |  |  |
| Fisher's Exact Test |  |  |  | <,001 | <,001 |
| Linear-by-Linear Association | 313,892 | 1 | <,001 |  |  |
| N of Valid Cases | 1006 |  |  |  |  |
| a. 0 cells (0,0%) have expected count less than 5. The minimum expected count is 24,24. | | | | | |
| b. Computed only for a 2x2 table | | | | | |

**Table S28. Elevated Blood glucose * Old or New Diabetes case Crosstabulation**

**Table S28a. Count**

|  | | Old or New Diabetes case | | Total |
| --- | --- | --- | --- | --- |
|  |  | Known case | New case |  |
| Elevated Blood glucose | No | 97 | 0 | 97 |
|  | Yes | 616 | 163 | 779 |
| Total | | 713 | 163 | 876 |

**Table S28b. Chi-Square Tests**

|  | Value | df | Asymptotic Significance (2-sided) | Exact Sig. (2-sided) | Exact Sig. (1-sided) |
| --- | --- | --- | --- | --- | --- |
| Pearson Chi-Square | 24,937^a^ | 1 | <,001 |  |  |
| Continuity Correction^b^ | 23,574 | 1 | <,001 |  |  |
| Likelihood Ratio | 42,620 | 1 | <,001 |  |  |
| Fisher's Exact Test |  |  |  | <,001 | <,001 |
| Linear-by-Linear Association | 24,908 | 1 | <,001 |  |  |
| N of Valid Cases | 876 |  |  |  |  |

**Table S29. Custom Tables**

|  |  | Age | | | Blood Glucose/ mg/dl | | | Systolic BP (mmHg) | | | Diastolic BP (mmHg) | | | Waist circumference (Centimeters) | | | Weight (Kg) | | | Body Mass Index (kg/m²) | | |
| --- | --- | --- | --- | --- | --- | --- | --- | --- | --- | --- | --- | --- | --- | --- | --- | --- | --- | --- | --- | --- | --- | --- |
|  |  | n | Mean | SD | n | Mean | SD | n | Mean | SD | n | Mean | SD | n | Mean | SD | n | Mean | SD | n | Mean | SD |
| Gender | Female | 2417 | 45.7 | 15.9 | 2417 | 101.2 | 22.0 | 2417 | 125.9 | 18.9 | 2417 | 75.5 | 10.7 | 2417 | 76.4 | 15.7 | 2417 | 75.7 | 10.7 | 2417 | 27.8 | 4.0 |
|  | Male | 1867 | 47.4 | 15.7 | 1867 | 100.5 | 21.3 | 1867 | 128.0 | 18.1 | 1867 | 75.8 | 10.9 | 1867 | 71.8 | 17.4 | 1867 | 70.8 | 5.9 | 1867 | 25.1 | 2.4 |
| Aged 45 years or more | Aged of less than 45 years | 2115 | 33.1 | 6.8 | 2115 | 100.1 | 22.1 | 2115 | 121.5 | 15.0 | 2115 | 73.0 | 9.9 | 2115 | 71.6 | 17.5 | 2115 | 72.9 | 9.1 | 2115 | 26.5 | 3.6 |
|  | Aged of 45 years or more | 2169 | 59.5 | 10.2 | 2169 | 101.7 | 21.2 | 2169 | 132.0 | 20.2 | 2169 | 78.2 | 10.9 | 2169 | 77.1 | 15.2 | 2169 | 74.3 | 9.4 | 2169 | 26.9 | 3.7 |
| Smoking | Never smoke | 2827 | 41.4 | 14.1 | 2827 | 99.7 | 20.7 | 2827 | 123.6 | 16.2 | 2827 | 74.1 | 9.9 | 2827 | 72.5 | 16.5 | 2827 | 72.6 | 8.5 | 2827 | 26.3 | 3.4 |
|  | Ex-smoker | 821 | 57.3 | 14.1 | 821 | 100.2 | 19.9 | 821 | 127.9 | 19.2 | 821 | 76.1 | 10.8 | 821 | 74.5 | 14.5 | 821 | 72.7 | 8.2 | 821 | 26.3 | 3.4 |
|  | Current smoker | 636 | 54.8 | 14.5 | 636 | 107.1 | 26.5 | 636 | 139.9 | 21.7 | 636 | 81.9 | 11.8 | 636 | 82.8 | 16.9 | 636 | 79.1 | 11.6 | 636 | 28.6 | 4.6 |
| Smoking duration | NA | 3057 | 42.3 | 14.4 | 3057 | 99.9 | 21.0 | 3057 | 123.8 | 16.4 | 3057 | 74.2 | 10.0 | 3057 | 72.6 | 16.4 | 3057 | 72.7 | 8.5 | 3057 | 26.3 | 3.4 |
|  | 0-5 years | 238 | 52.4 | 14.6 | 238 | 102.2 | 23.7 | 238 | 129.4 | 18.5 | 238 | 76.3 | 10.4 | 238 | 75.6 | 15.0 | 238 | 74.9 | 9.7 | 238 | 27.3 | 4.0 |
|  | 6-10 years | 162 | 53.3 | 14.0 | 162 | 102.0 | 20.3 | 162 | 138.5 | 22.0 | 162 | 81.3 | 12.1 | 162 | 80.7 | 17.0 | 162 | 76.2 | 11.4 | 162 | 27.7 | 4.4 |
|  | 11-15 years | 58 | 51.4 | 13.5 | 58 | 99.8 | 17.5 | 58 | 124.8 | 16.9 | 58 | 75.4 | 11.1 | 58 | 72.3 | 15.5 | 58 | 71.3 | 7.7 | 58 | 25.5 | 3.1 |
|  | > 15 years | 769 | 59.3 | 13.7 | 769 | 104.2 | 23.9 | 769 | 135.9 | 21.9 | 769 | 80.0 | 11.8 | 769 | 79.8 | 16.2 | 769 | 76.5 | 10.8 | 769 | 27.7 | 4.3 |
| Alcohol intake | Never drink | 1257 | 39.4 | 13.8 | 1257 | 100.6 | 21.3 | 1257 | 122.1 | 15.9 | 1257 | 73.4 | 9.9 | 1257 | 71.5 | 16.9 | 1257 | 73.1 | 8.6 | 1257 | 26.6 | 3.4 |
|  | Ex-drinker | 1262 | 49.8 | 15.8 | 1262 | 98.8 | 19.8 | 1262 | 125.3 | 18.2 | 1262 | 74.7 | 10.3 | 1262 | 74.7 | 15.3 | 1262 | 72.7 | 8.6 | 1262 | 26.4 | 3.4 |
|  | Drink occasionally | 1159 | 48.6 | 15.7 | 1159 | 101.0 | 22.4 | 1159 | 129.5 | 19.4 | 1159 | 77.1 | 11.1 | 1159 | 75.6 | 16.6 | 1159 | 74.0 | 9.7 | 1159 | 26.8 | 3.8 |
|  | Daily 1 drink for woman or 1-2 drinks for man | 195 | 47.7 | 14.8 | 195 | 104.3 | 23.6 | 195 | 120.7 | 11.5 | 195 | 72.0 | 8.1 | 195 | 72.7 | 15.2 | 195 | 71.8 | 7.9 | 195 | 25.8 | 3.3 |
|  | Daily 2 drinks for woman or 3 drinks for man | 123 | 47.8 | 14.1 | 123 | 100.2 | 16.0 | 123 | 126.4 | 13.2 | 123 | 76.0 | 9.5 | 123 | 72.2 | 15.5 | 123 | 72.0 | 8.1 | 123 | 25.7 | 3.1 |
|  | Daily 3 drinks for woman or 4 drinks for man | 33 | 48.0 | 11.7 | 33 | 101.3 | 18.9 | 33 | 131.0 | 16.2 | 33 | 78.8 | 11.4 | 33 | 73.4 | 16.2 | 33 | 68.7 | 5.5 | 33 | 24.2 | 1.9 |
|  | Daily > 3 drinks for woman or > 4 drinks for man | 255 | 53.1 | 15.8 | 255 | 109.4 | 26.7 | 255 | 149.1 | 17.5 | 255 | 85.9 | 10.7 | 255 | 84.0 | 18.6 | 255 | 80.7 | 12.1 | 255 | 29.2 | 4.8 |
| Alcohol duration | NA | 1552 | 41.0 | 14.5 | 1552 | 99.8 | 20.4 | 1552 | 121.8 | 15.2 | 1552 | 73.2 | 9.7 | 1552 | 72.2 | 16.3 | 1552 | 72.9 | 8.4 | 1552 | 26.5 | 3.3 |
|  | 0-5 years | 537 | 42.8 | 15.3 | 537 | 98.9 | 20.7 | 537 | 126.7 | 19.1 | 537 | 75.4 | 11.0 | 537 | 73.5 | 17.5 | 537 | 73.6 | 9.8 | 537 | 26.7 | 3.8 |
|  | 6-10 years | 317 | 45.1 | 14.4 | 317 | 100.1 | 22.0 | 317 | 128.2 | 17.8 | 317 | 76.2 | 11.3 | 317 | 76.3 | 17.0 | 317 | 74.8 | 10.3 | 317 | 27.1 | 4.1 |
|  | 11-15 years | 234 | 48.2 | 16.1 | 234 | 106.2 | 26.1 | 234 | 133.8 | 20.4 | 234 | 78.5 | 11.2 | 234 | 80.4 | 17.1 | 234 | 77.7 | 11.2 | 234 | 28.3 | 4.6 |
|  | > 15 years | 1644 | 52.7 | 15.1 | 1644 | 101.9 | 22.2 | 1644 | 130.3 | 20.0 | 1644 | 77.4 | 11.0 | 1644 | 75.5 | 16.0 | 1644 | 73.4 | 9.1 | 1644 | 26.5 | 3.6 |
| Last food intake | ≥ 6 hours | 2494 | 48.6 | 15.7 | 2494 | 97.5 | 18.4 | 2494 | 127.5 | 19.0 | 2494 | 76.2 | 10.7 | 2494 | 75.3 | 16.2 | 2494 | 73.9 | 9.5 | 2494 | 26.8 | 3.7 |
|  | 3-6 hours | 1164 | 43.5 | 15.3 | 1164 | 102.0 | 20.2 | 1164 | 125.5 | 17.8 | 1164 | 75.1 | 10.5 | 1164 | 72.8 | 17.0 | 1164 | 73.0 | 8.9 | 1164 | 26.5 | 3.6 |
|  | 1-3Hrs | 614 | 43.1 | 15.7 | 614 | 110.8 | 29.0 | 614 | 126.6 | 18.1 | 614 | 74.2 | 11.1 | 614 | 73.5 | 17.1 | 614 | 73.2 | 9.0 | 614 | 26.5 | 3.6 |
|  | < 1 hour | 12 | 42.4 | 12.3 | 12 | 181.1 | 28.0 | 12 | 124.3 | 24.1 | 12 | 71.2 | 13.3 | 12 | 78.4 | 17.5 | 12 | 72.5 | 10.7 | 12 | 26.8 | 4.2 |
| Elevated Blood Pressure (SBP >= 140 and/or DBP >= 90) | No | 3374 | 44.1 | 15.2 | 3374 | 99.8 | 20.6 | 3374 | 119.5 | 11.8 | 3374 | 72.2 | 8.3 | 3374 | 72.8 | 15.6 | 3374 | 72.8 | 8.2 | 3374 | 26.4 | 3.3 |
|  | Yes | 910 | 55.0 | 15.0 | 910 | 104.7 | 24.8 | 910 | 153.8 | 13.7 | 910 | 88.3 | 9.0 | 910 | 80.4 | 18.6 | 910 | 76.6 | 12.0 | 910 | 27.7 | 4.7 |
| Old or New HTN case | Known case | 254 | 56.2 | 14.9 | 254 | 105.1 | 30.2 | 254 | 145.4 | 16.9 | 254 | 84.1 | 10.3 | 254 | 77.5 | 17.4 | 254 | 74.5 | 10.8 | 254 | 26.9 | 4.2 |
|  | New case | 752 | 54.8 | 15.1 | 752 | 105.0 | 23.7 | 752 | 153.8 | 13.1 | 752 | 88.3 | 9.1 | 752 | 80.5 | 18.6 | 752 | 76.7 | 12.0 | 752 | 27.7 | 4.7 |
| Elevated Blood glucose | No | 3505 | 45.2 | 15.5 | 3505 | 97.3 | 20.2 | 3505 | 126.0 | 18.0 | 3505 | 75.1 | 10.6 | 3505 | 73.6 | 16.3 | 3505 | 73.0 | 8.8 | 3505 | 26.5 | 3.5 |
|  | Yes | 779 | 51.9 | 16.2 | 779 | 117.1 | 20.7 | 779 | 130.4 | 20.5 | 779 | 77.8 | 11.1 | 779 | 78.0 | 17.5 | 779 | 76.1 | 10.6 | 779 | 27.6 | 4.2 |
| Old or New Diabetes case | Known case | 713 | 49.9 | 16.4 | 713 | 117.4 | 22.4 | 713 | 129.9 | 20.5 | 713 | 77.3 | 11.1 | 713 | 77.4 | 17.5 | 713 | 74.3 | 11.4 | 713 | 27.1 | 4.3 |
|  | New case | 163 | 52.4 | 16.1 | 163 | 117.4 | 24.5 | 163 | 127.3 | 18.2 | 163 | 76.1 | 10.7 | 163 | 76.7 | 17.8 | 163 | 76.7 | 9.3 | 163 | 27.3 | 4.1 |
